# Temporal transcriptomic changes in microRNAs involved in the host immune response and metabolism during *Neospora caninum* infection

**DOI:** 10.1186/s13071-023-05665-9

**Published:** 2023-01-24

**Authors:** Jin-Ming Chen, Shan-Shan Zhao, De-Liang Tao, Jing-Yu Li, Xin Yang, Ying-Ying Fan, Jun-Ke Song, Qun Liu, Guang-Hui Zhao

**Affiliations:** 1 Department of Parasitology, College of Veterinary Medicine, Northwest A&F University, Yangling, 712100 China; 2 National Animal Protozoa Laboratory, College of Veterinary Medicine, China Agricultural University, Beijing, 100193 China

**Keywords:** *Neospora caninum*, Caprine endometrial epithelial cells, MicroRNA, Expression, RNA sequencing

## Abstract

**Background:**

*Neospora caninum* infection is a major cause of abortion in cattle, which results in serious economic losses to the cattle industry. However, there are no effective drugs or vaccines for the control of *N. caninum* infections. There is increasing evidence that microRNAs (miRNAs) are involved in many physiological and pathological processes, and dysregulated expression of host miRNAs and the biological implications of this have been reported for infections by various protozoan parasites. However, to our knowledge, there is presently no published information on host miRNA expression during *N. caninum* infection.

**Methods:**

The expression profiles of miRNAs were investigated by RNA sequencing (RNA-seq) in caprine endometrial epithelial cells (EECs) infected with *N. caninum* at 24 h post infection (pi) and 48 hpi, and the functions of differentially expressed (DE) miRNAs were predicted by Gene Ontology (GO) and Kyoto Encyclopedia of Genes and Genomes (KEGG) enrichment analyses. The transcriptome data were validated by using quantitative real-time polymerase chain reaction. One of the upregulated DEmiRNAs, namely chi-miR-146a, was selected to study the effect of DEmiRNAs on the propagation of *N. caninum* tachyzoites in caprine EECs.

**Results:**

RNA-seq showed 18 (17 up- and one downregulated) and 79 (54 up- and 25 downregulated) DEmiRNAs at 24 hpi and 48 hpi, respectively. Quantitative real-time polymerase chain reaction analysis of 13 randomly selected DEmiRNAs (10 up- and three downregulated miRNAs) confirmed the validity of the RNA-seq data. A total of 7835 messenger RNAs were predicted to be potential targets for 66 DEmiRNAs, and GO and KEGG enrichment analysis of these predicted targets revealed that DEmiRNAs altered by *N. caninum* infection may be involved in host immune responses (e.g. Fc gamma R-mediated phagocytosis, Toll-like receptor signaling pathway, tumor necrosis factor signaling pathway, transforming growth factor-β signaling pathway, mitogen-activated protein kinase signaling pathway) and metabolic pathways (e.g. lysine degradation, insulin signaling pathway, AMP-activated protein kinase signaling pathway, Rap1 signaling pathway, calcium signaling pathway). Upregulated chi-miR-146a was found to promote *N. caninum* propagation in caprine EECs.

**Conclusions:**

This is, to our knowledge, the first report on the expression profiles of host miRNAs during infection with *N. caninum*, and shows that chi-miR-146a may promote *N. caninum* propagation in host cells. The novel findings of the present study should help to elucidate the interactions between host cells and *N. caninum*.

**Graphical Abstract:**

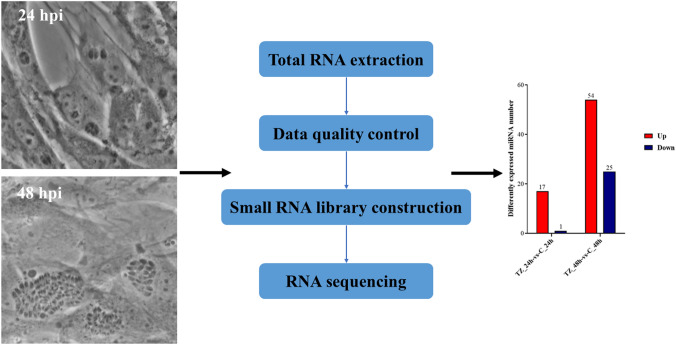

**Supplementary Information:**

The online version contains supplementary material available at 10.1186/s13071-023-05665-9.

## Background

*Neospora caninum* is an important intracellular protozoan parasite that infects a variety of animals, including domesticated ruminants (e.g. cattle, sheep, goats) and wildlife [1, 2]. Infection with *N. caninum* has been reported to cause reproductive disorders in pregnant animals and neuromuscular disorders in newborn animals [3, 4]. Notably, neosporosis, which is caused by *N. caninum* infection, was found to be one of the main causes of abortion in cattle [5, 6]. A meta-analysis also found that the combined seroprevalence of *N. caninum* infection in goats was 5.99%, and seropositive goats were more likely to abort than seronegative ones [7]. Although there is insufficient evidence to indicate that *N. caninum* is of zoonotic significance, antibodies against *N. caninum* have been detected in humans [8]. However, no effective drugs or vaccines have yet been developed to control neosporosis or infection with *N. caninum* [9–12]. At present, eliminating cattle positive for antibodies against *N. caninum* is the most common strategy used in cattle production against this parasite [13, 14]. This results in huge economic losses, with a total annual loss of more than $1 billion to the cattle industry of 10 countries alone [15].

MicroRNAs (miRNAs) are a type of endogenous non-coding RNA (ncRNA), approximately 19–25 nucleotides (nt) in length [16]. In studies by Lei [17] and Bartel [18], miRNA expression was generally dysregulated, and miRNAs were found to function as key elements in the regulation of target mRNAs through their combination with the 3' untranslated region of the latter. They have also been found to play a role in translational inhibition and/or mRNA degradation at post-transcriptional levels [19] in a great number of biological processes (e.g. cell proliferation [20], apoptosis [21, 22], autophagy [23] and pyroptosis [24]), and during the progression of various diseases [25, 26], including infections with protozoan parasites. For example, a total of 81, 126, 82 and 131 miRNAs were differentially expressed (DE) in porcine alveolar macrophages [27], pig splenocytes [28], livers of domestic cats [29], and mouse spleen [30], respectively, during infections with *Toxoplasma gondii*, an obligate intracellular zoonotic protozoan parasite similar to *N. caninum* in its morphological and biological features but differing in its life cycle and biological effects on host cells [31–33]. Previously, our group found that *N. caninum* infection significantly altered the expression patterns of host cell long ncRNAs, which are some of the most important regulatory ncRNAs [34]. In the present study, we investigated dynamic miRNA profiles in caprine endometrial epithelial cells (EECs) during their infections with *N. caninum*.

## Methods

### Cells

African green monkey kidney cells (Vero cells) and caprine EECs were provided by Prof. Xuefeng Qi and Prof. Yaping Jin from Northwest A&F University (Shaanxi, China), respectively. Vero cells were cultured in DMEM medium supplemented with 10% fetal bovine serum (FBS; ExCell Bio, Shanghai, China), and caprine EECs were cultured in DMEM/F12 medium supplemented with 10% FBS. All cells were cultured with 5% CO_2_ at 37 ℃ in a CO_2_ incubator (Thermo Fisher Scientific, Wilmington, NC).

### Parasite purification and infection

NC-1 N*. caninum* tachyzoites were gifted by Prof. Qun Liu from the China Agricultural University (Beijing, China), and passaged in Vero cells in DMEM medium supplemented with 2% FBS, 100 U/ml penicillin, and 100 μg/ml streptomycin. Before infection, Vero cells infected with tachyzoites were scraped, pipetted using a 27-gauge needle, filtered through a 5.0-µm filter, and centrifuged at 716 g for 10 min. After the supernatant had been discarded, the pellets were resuspended in fresh medium for further study, and the number of tachyzoites was counted with a hemocytometer.

The in vitro infection model in caprine EECs was established in accordance with our previous study [35] at a multiplicity of infection of 3:1 (tachyzoites:cells).

### Sample preparation and RNA extraction

Caprine EECs infected (experimental group) or not infected (control group) with *N. caninum* tachyzoites were collected at 24 h (experimental groups, TZ1_24h to TZ3_24h; control groups, C1_24h to C3_24h) and 48 h (experimental groups, TZ1_48h to TZ3_48h; control groups, C1_48h to C3_48h) post-infection [pi; hours pi (hpi)], treated with Trizol reagent (Invitrogen, Carlsbad, CA), and stored at —80 °C until RNA extraction. All the analyses were performed with three biological replicates.

The total RNA for each sample was extracted by using a mirVana miRNA isolation kit (Ambion, Austin, TX) following the manufacturer’s protocol. The extracted RNA samples were quantified by using Nanodrop 2000 (Thermo Fisher Scientific). The RNA integrity of each RNA sample was checked using an Agilent 2100 Bioanalyzer (Agilent Technologies, CA), and RNA samples with 28S/18S ≥ 0.7 and an RNA integrity number ≥ 7 were used for further analysis.

### Small RNA library construction and RNA sequencing

A total of 1 μg RNA from each sample was used to construct the small RNA library by using TruSeq Small RNA Sample Prep Kits (Illumina, USA) following the manufacturer’s recommendations. Briefly, the total RNA samples were ligated to adapters at each end, and then the adapter-ligated RNA specimens were reverse transcribed into complementary DNA (cDNA) samples for polymerase chain reaction (PCR) amplification. PCR products ranging from 140 to 160 base pairs in length were purified as a small RNA library. The quality of the library was assessed on the Agilent Bioanalyzer 2100 system by using DNA high-sensitivity chips. These small RNA libraries were sequenced using the Illumina HiSeq X Ten platform to generate a 150-base pairs paired-end. All analyses were performed by OE Biotechnology (Shanghai, China).

### Bioinformatic analysis

The basic reads obtained by RNA sequencing (RNA-seq) were converted into raw reads by base calling. Adapter sequences of raw reads were removed by using cutadapt (version 1.14) [36], and sequences shorter than 15 nt and longer than 41 nt were filtered out. The obtained sequences were subjected to Q20 quality control by using fastx_toolkit (version 0.0.13) software (http://hannonlab.cshl.edu/fastx_toolkit), and the reads containing N bases were filtered out by using NGSQCToolkit (version 2.3.2) [37] to obtain high-quality clean reads for subsequent analysis.

The obtained clean reads were mapped to the reference genome from *Capra hircus* (ftp://ftp.ncbi.nlm.nih.gov/genomes/all/GCF/001/704/415/GCF_001704415.1_ ARS1/gcf_00-1,704,415.1_ars1_genome.fna.gz) and the percentage of these reads that were aligned to the genome counted. Using bowtie software [38], the clean reads were aligned with the Rfam v.10.0 database (http://www.sanger.ac.uk/software/Rfam) [39] to annotate ribosomal RNA, small nuclear RNA, small nucleolar RNA, and transfer RNA, and then the sequences that were annotated to the Rfam database were filtered and removed. The sequences aligned to the transcripts less than 15 nt and more than 26 nt in length were removed. Using Repeat Masker software [40], the filtered sequences were aligned to the repeat database to identify possible repetitive sequences and filtered. The identified miRNAs were aligned with the miRbase v.22.0 database (http://www.mirbase.org/) [41] to analyze the expression levels of the known miRNAs, and the unannotated small RNA sequences were analyzed by Mirdeep2 [42] to predict novel miRNAs, and the secondary structure of these novel miRNAs were predicted by using RNAfold software.

To analyze the expression levels of DEmiRNAs between the experimental group and the control group at 24 hpi and 48 hpi after *N. caninum* infection, the expression level of each sample was calculated using transcripts per million [43], and the differential expression was calculated by using DESeq2 1.16.1, with a *q*-value < 0.05 and | log_2_ fold change |> 0 considered as significantly different.

### Verification of miRNA expression by quantitative real-time PCR

Thirteen (10 up- and three downregulated) DEmiRNAs were randomly selected for quantitative real-time PCR (qRT-PCR) analysis to verify the sequencing data. A total of 12 samples from the experimental (six samples) and control (six samples) groups were collected at 24 hpi and 48 hpi, and the total RNA of each sample was extracted using Trizol reagent (Invitrogen, Carlsbad, CA). The quality of each RNA sample was evaluated by using a Nano-100 spectrophotometer (Hangzhou, China). The cDNA samples were synthesized using 0.8-μg RNA samples in a Mir-XTM miRNA First-Strand Synthesis Kit, in accordance with the manufacturer’s instructions. qRT-PCR reactions were performed in a 25-μl mixture containing 2 μl cDNA, 9 μl ddH_2_O, 0.5 μl ROX reference dye, 0.5 μl miRNA-specific forward primer, 0.5 μl mRQ 3' primer, and 12.5 μl TB Green Premix *Ex Taq*TM II (Tli RNaseH Plus) under the following conditions: 95 ℃ for 10 min, 40 cycles of 95 ℃ for 15 s, 55–58 ℃ (Additional file 1: Data S1) for 30 s, in a Four-channel Real-time Fluorescence Quantitative PCR system (Tianlong TL988, Shaanxi, China). Three biological replicates were performed for each reaction, and the *u6* small nuclear RNA gene was used as the expression level standard. The relative expression of each gene was calculated by using the 2^−ΔΔCt^ method, with *P* < 0.05 indicating statistically significant difference.

### Target prediction and function analysis of DEmiRNAs

Targets of all DEmiRNAs were predicted by using miranda software [44] with *S* ≥ 150, Δ*G* ≤  − 30 kcal/mol and strict 5’ seed pairing. Functions of the DEmiRNAs were predicted by submitting their targets to Gene Ontology (GO) and Kyoto Encyclopedia of Genes and Genomes (KEGG) databases for enrichment analysis, using R based on the hypergeometric distribution. GO enrichment analysis included three elements: biological process (BP), cellular composition (CC) and molecular function (MF). KEGG enrichment analysis was used to identify the pathways and predict biological functions. Elements with a *q*-value < 0.05 were considered to be significantly enriched.

### Propagation of *N. caninum* tachyzoites affected by DEmiRNAs

To study the effect of DEmiRNAs on the propagation of *N. caninum* tachyzoites in caprine EECs, one upregulated miRNA, namely chi-miR-146a, was selected. The mimics and inhibitor of chi-miR-146a together with their negative controls were obtained from GenePharma (Shanghai, China). First, a total of 100 pmol of mimics or inhibitor was transfected into caprine EECs by using Lipofectamine 2000 reagent (Invitrogen, Gaithersburg, MD). Then, *N. caninum* tachyzoites were infected at 24 h post-transfection with a multiplicity of infection of 3:1 (tachyzoites:cells), and the number of parasites per vacuole was determined at 30 hpi or 42 hpi in 100 parasitophorous vacuoles under fluorescence microscopy (Olympus, Tokyo, Japan).

### Statistical analysis

The differences between the control and the experimental groups were analyzed by using GraphPad Prism 5.0 software (http://www.graphpad.com), and *P* < 0.05 was considered to indicate a statistically significant difference in the two-tailed *t*-test, which was used as the parametric test.

## Results

### Identification of caprine miRNAs

A total of 343.84 M raw reads were generated from 12 samples by RNA-seq. Through QC quality control (adapter sequences, N base sequence and Q20), a total of 294.44 M clean reads and 15.77 M unique reads were obtained (Table 1), and 75.10% (221112855) of these clean reads (294429204) were matched to the reference genome of *C. hircus* (Additional file 2: Data S2). Through Rfam alignment, transcript sequence alignment, repeat sequence alignment and miRNA (miRBase database) alignment annotation, 348 known and 891 novel predicted miRNAs were identified (Additional file 3: Data S3).

**Table 1:** Statistics of the clean reads of the small RNA libraries

Sample	Raw reads	Trimmed length	Trimmed Q20	Trimmed N	Clean reads	Unique reads
C1_24h	29.53 M	24.97 M	24.95 M	24.95 M	24.95 M	0.77 M
C1_48h	28.70 M	24.85 M	24.83 M	24.83 M	24.83 M	0.76 M
C2_24h	27.34 M	24.37 M	24.36 M	24.35 M	24.35 M	0.76 M
C2_48h	27.90 M	24.67 M	24.65 M	24.65 M	24.65 M	0.71 M
C3_24h	28.35 M	24.74 M	24.73 M	24.73 M	24.73 M	0.77 M
C3_48h	27.70 M	24.51 M	24.48 M	24.48 M	24.48 M	0.65 M
TZ1_24h	29.15 M	24.19 M	24.17 M	24.17 M	24.17 M	1.79 M
TZ1_48h	29.68 M	24.79 M	24.77 M	24.77 M	24.77 M	1.99 M
TZ2_24h	27.92 M	24.02 M	24.00 M	23.99 M	23.99 M	1.75 M
TZ2_48h	28.53 M	24.23 M	24.21 M	24.21 M	24.21 M	2.11 M
TZ3_24h	28.98 M	24.86 M	24.85 M	24.84 M	24.84 M	1.92 M
TZ3_48h	30.06 M	24.48 M	24.47 M	24.47 M	24.47 M	1.79 M

### Differential expression profiles of miRNAs in caprine EECs infected with *N. caninum*

**Fig. 1:**
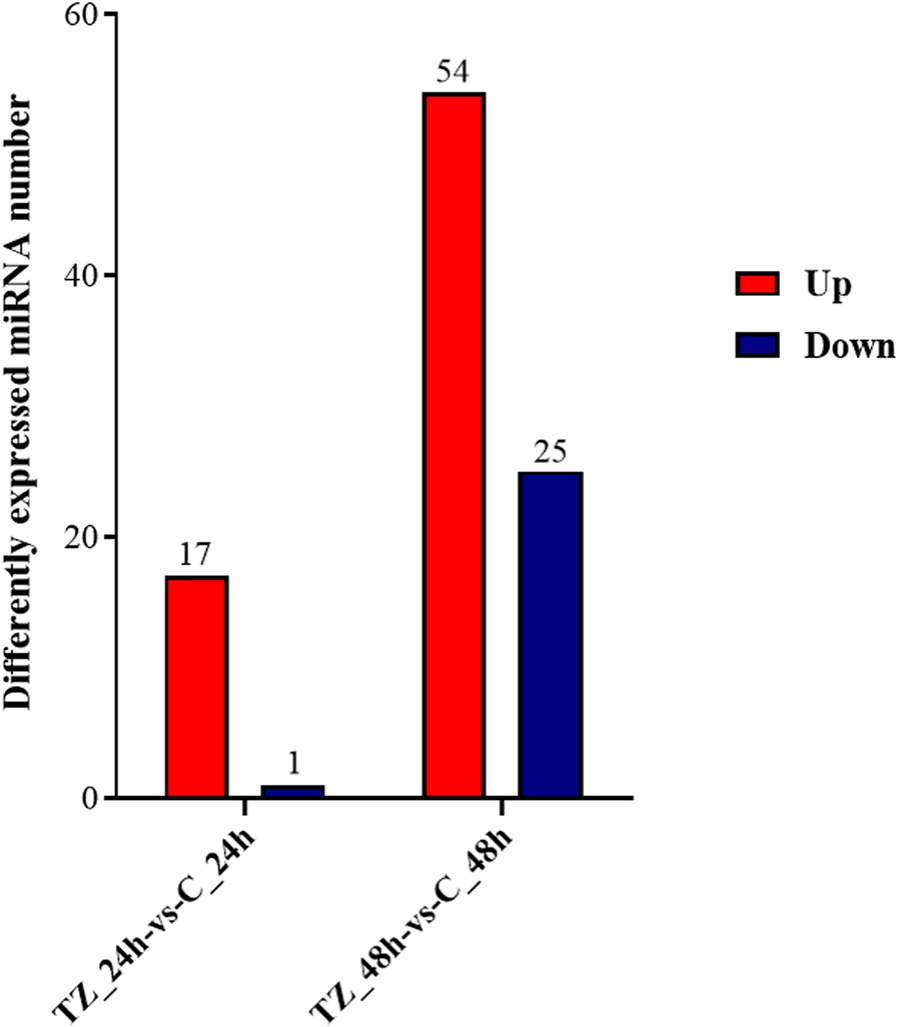
The number of differently expressed (DE) microRNAs (miRNAs)

**Fig. 2:**
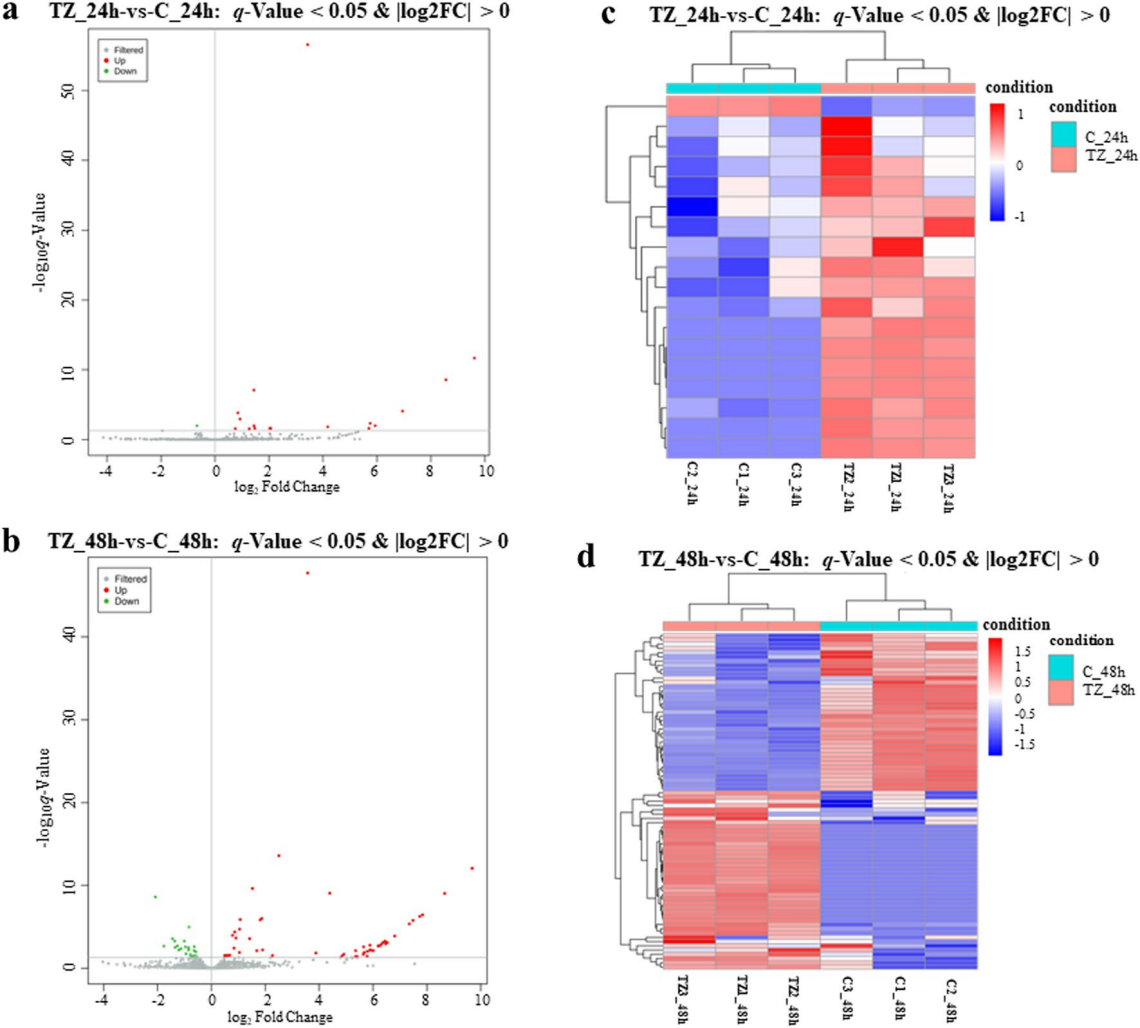
**a**–**d** The expression patterns of DEmiRNAs in caprine endometrial epithelial cells (EECs) infected with *Neospora caninum*. **a**, **b** The volcano plot of the miRNA distributions. Gray dots represent non-differential miRNAs, red dots represent significantly upregulated miRNAs, and green dots represent significantly downregulated miRNAs. **c**, **d** The hierarchical cluster plot of the miRNAs expression profiles for the categories TZ_24h-vs-C_24h (**c**) and TZ_48h-vs-C_48h (**d**). C_24 h/48 h are control groups and TZ_24 h/48 h are experimental groups (*q*-value < 0.05 and | log_2_fold change |> 0)

To analyze the differential expression profiles of miRNAs in caprine EECs during *N. caninum* infection, two categories were used for comparison, namely TZ_24h-vs-C_24h and TZ_48h-vs-C_48h. When using the criteria of a *q*-value < 0.05 and | log_2_fold change |> 0, a total of 84 (59 up- and 25 downregulated) DEmiRNAs were found to be dysregulated, with 13 (12 up- and one downregulated) miRNAs differentially expressed in both of the categories (TZ_24h-vs-C_24h and TZ_48h-vs-C_48h). Notably, five of the upregulated miRNAs were only found in category TZ_24h-vs-C_24h, while 66 (42 up- and 24 downregulated) DEmiRNAs were detected in category TZ_48h-vs-C_48h (Fig. 1). The volcano maps (Fig. 2a, b) show the overall distribution of the DEmiRNAs, and the hierarchical clustering heatmaps (Fig. 2c, d) clearly differentiate the control and experimental groups. Detailed information on the DEmiRNAs is given in Additional file 4: Data S4.

### Validation of DEmiRNAs by qRT-PCR

**Fig. 3:**
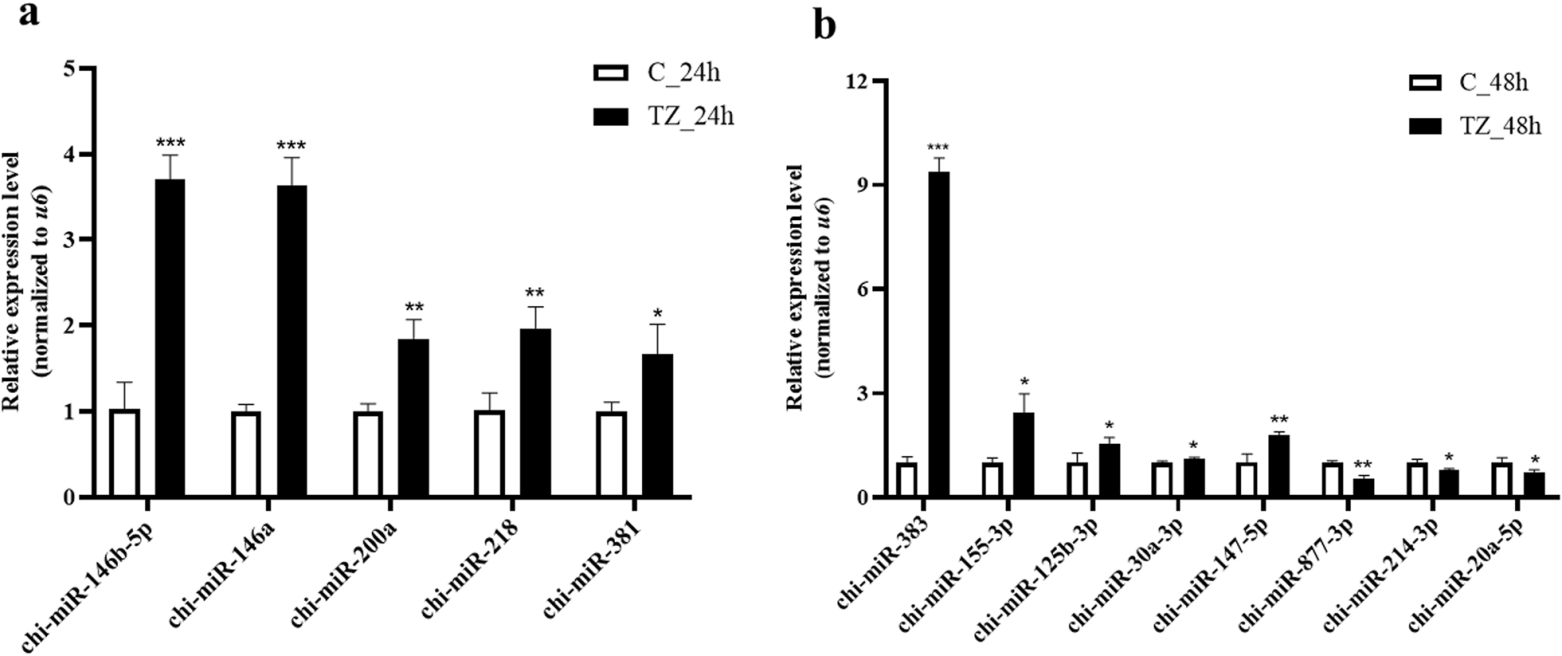
**a**, **b** Validation of the DEmiRNAs by using quantitative real-time PCR. **a**, **b** Validation results for miRNAs in the categories TZ_24h-vs-C_24h (**a**) and TZ_48h-vs-C_48h (**b**), with three biological repeats included for each miRNA. * *P* < 0.05, ** *P* < 0.01, *** *P* < 0.001

To validate the accuracy of the RNA-seq data, five upregulated (chi-miR-146b-5p, chi-miR-146a, chi-miR-200a, chi-miR-218, chi-miR-381) DEmiRNAs were selected for qRT-PCR analysis from category TZ_24h-vs-C_24h (Fig. 3a). Five up- (chi-miR-383, chi-miR-155-3p, chi-miR-125b-3p, chi-miR-30a-3p, chi-miR-147-5p) and three downregulated (chi-miR-877-3p, chi-miR-214-3p, chi-miR-20a-5p) DEmiRNAs were selected for qRT-PCR analysis from category TZ_48h-vs-C_48h (Fig. 3b). The expression of all the determined DEmiRNAs was consistent with the RNA-seq data, indicating high credibility of the latter.

### Target prediction and functional annotation of DEmiRNAs

**Fig. 4:**
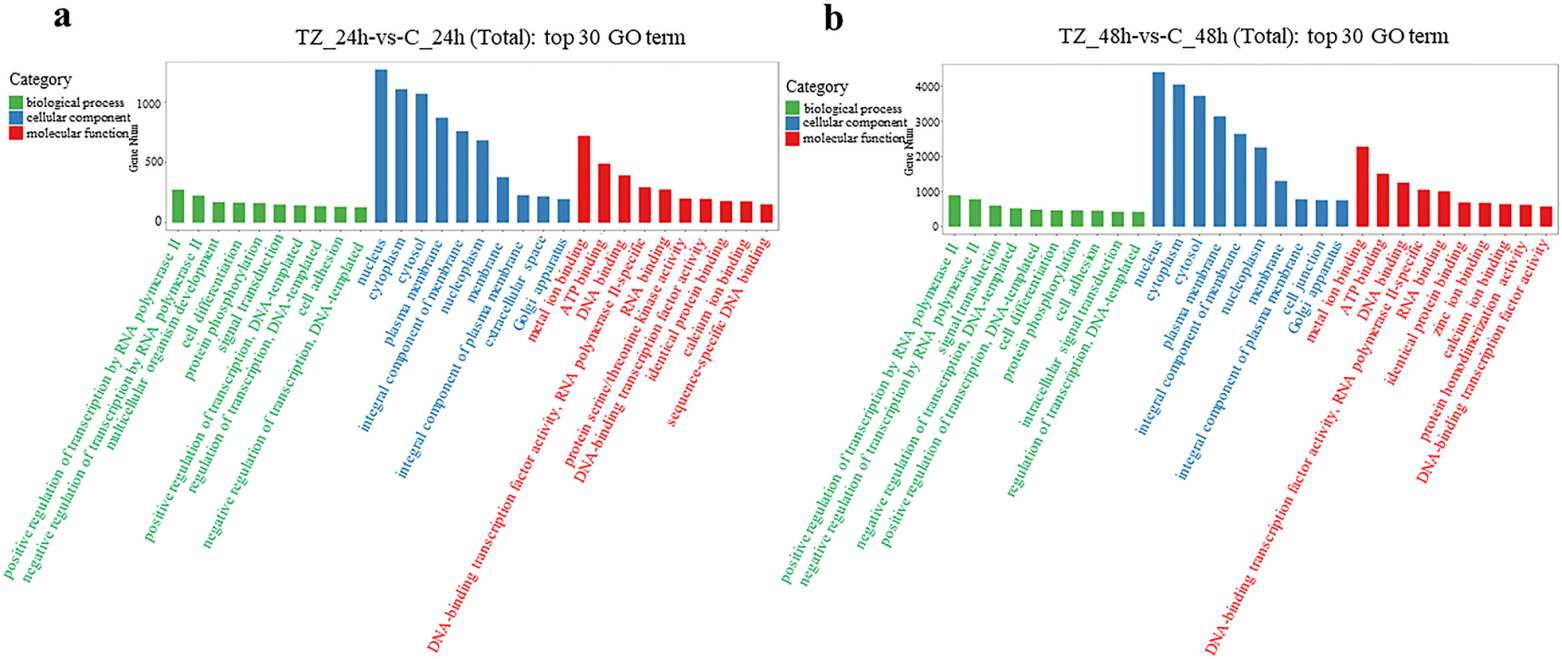
**a**, **b** Gene Ontology (*GO*) enrichment analysis of targets for the DEmiRNAs in caprine EECs during *Neospora caninum* infection. **a**, **b** The predicted top 30 GO terms targeted by DEmiRNAs in caprine EECs infected with *N. caninum* in the categories TZ_24h-vs-C_24h (**a**) and TZ_48h-vs-C_48h (**b**)

A total of 7835 mRNAs were predicted to be potential targets for 66 DEmiRNAs by miranda software, and comprised 2094 and 7712 mRNAs for 12 and 63 DEmiRNAs at 24 hpi and 48 hpi, respectively (Additional file 5: Data S5). GO analysis of these targets indicated that 883 and 1752 terms were significantly enriched for the categories TZ_24h-vs-C_24h and TZ_48h-vs-C_48h, respectively (Additional file 6: Data S6). The top 30 significantly enriched terms are shown in Fig. 4. Interestingly, most of the significantly enriched terms in BP, CC and MF were the same for the two categories, while only one, one and two terms in BP, CC and MF, respectively, differed between the two categories.

**Fig. 5:**
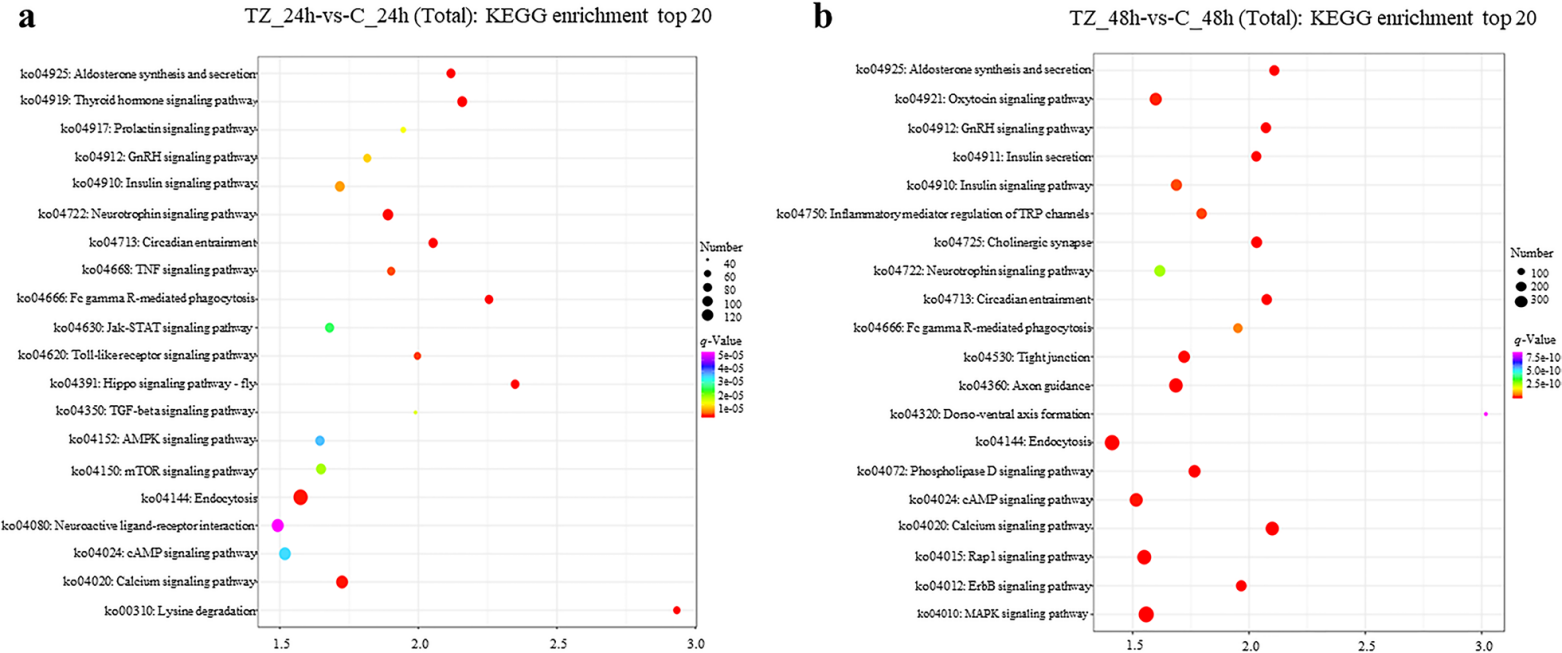
**a**, **b** Kyoto Encyclopedia of Genes and Genomes (*KEGG*) enrichment analysis of targets for the DEmiRNAs in caprine EECs during *Neospora caninum* infection. **a**, **b** The predicted top 20 KEGG terms targeted by DEmiRNAs in caprine EECs infected with *N. caninum* in the categories TZ_24h-vs-C_24h (**a**) and TZ_48h-vs-C_48h (**b**)

KEGG enrichment analysis of targets for DEmiRNAs showed that 42 and 90 terms were significantly enriched for the categories TZ_24h-vs-C_24h and TZ_48h-vs-C_48h, respectively (Additional file 7: Data S7). The top 20 significantly enriched terms are shown in Fig. 5. Surprisingly, in the KEGG enrichment analysis, contrary to the results of the GO analysis, most of the terms that were significantly enriched differed between the two categories. For example, several pathways involved in signal transduction [e.g. Hippo signaling pathway—fly, tumor necrosis factor (TNF) signaling pathway, transforming growth factor (TGF)-β signaling pathway, mTOR signaling pathway, Jak-STAT signaling pathway, AMP-activated protein kinase (AMPK) signaling pathway], amino acid metabolism (e.g. lysine degradation), and signaling molecules and interaction (e.g. neuroactive ligand-receptor interaction) were significantly enriched in the category TZ_24h-vs-C_24h, while some pathways involved in other signal transduction pathways [e.g. mitogen-activated protein kinase (MAPK) signaling pathway, Rap1 signaling pathway, phospholipase D signaling pathway, ErbB signaling pathway], and development and regeneration (e.g. axon guidance, dorso-ventral axis formation) were significantly enriched in the category TZ_48h-vs-C_48h. Of these, most of the targets were predicted to be involved in immune (e.g. Hippo signaling pathway—fly, Fc gamma R-mediated phagocytosis, Toll-like receptor signaling pathway, TNF signaling pathway, TGF-β signaling pathway, mTOR signaling pathway, Jak-STAT signaling pathway, MAPK signaling pathway, ErbB signaling pathway, inflammatory mediator regulation of TRP channels) and metabolic (e.g. lysine degradation, insulin signaling pathway, AMPK signaling pathway, Rap1 signaling pathway, calcium signaling pathway) pathways.

### Propagation of *N. caninum* tachyzoites in caprine EECs affected by chi-miR-146a

**Fig. 6:**
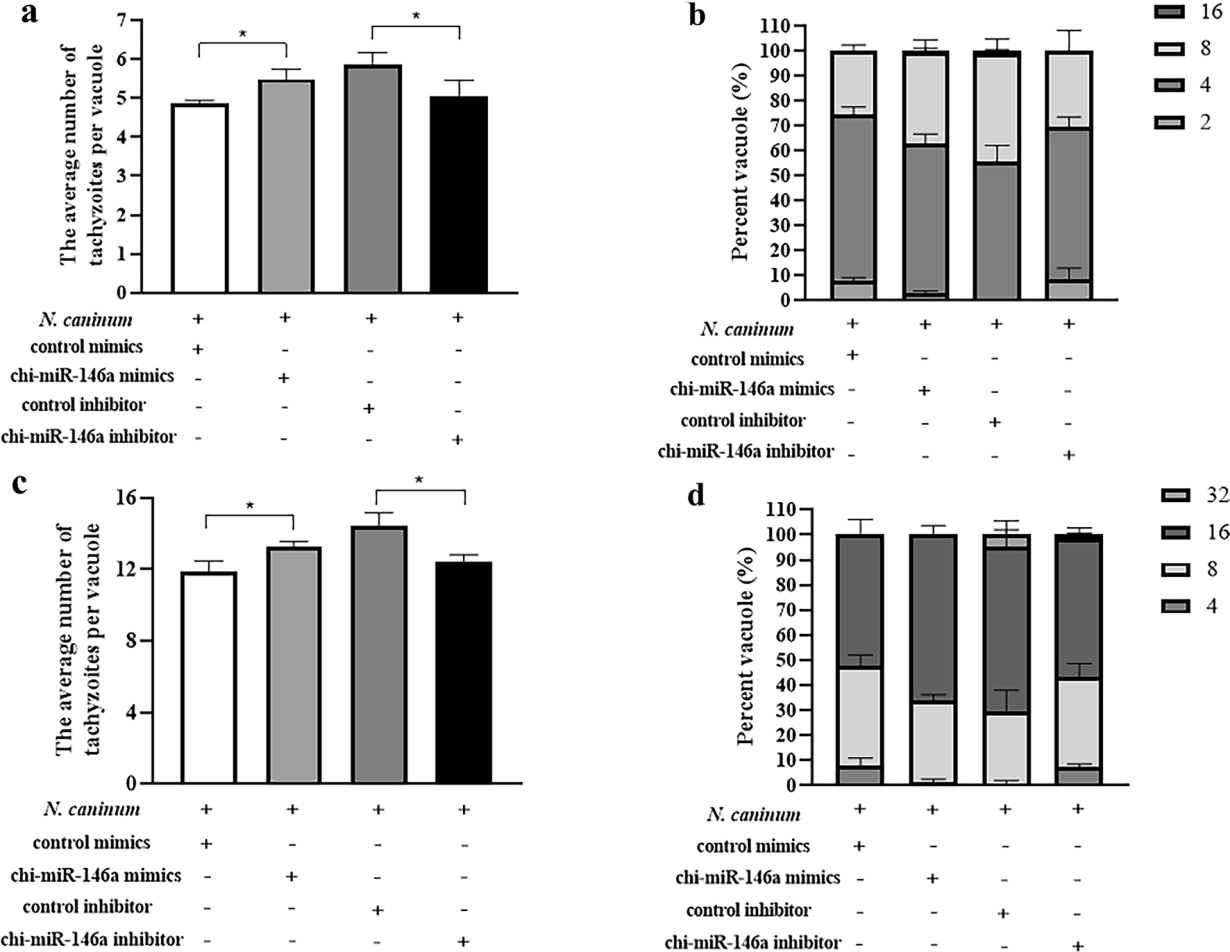
**a**–**d** Effect of chi-miR-146a on the propagation of *Neospora caninum* tachyzoites in caprine EECs. **a**, **b** The effect of chi-miR-146a on the propagation of *Neospora caninum* tachyzoites in caprine EECs at 30 h post-infection (hpi). **c**, **d** The effect of chi-miR-146a on the propagation of *N. caninum* tachyzoites in caprine EECs at 42 hpi. The average number of tachyzoites in 100 parasitophorous vacuoles at 30 hpi (**a**) and 42 hpi (**c**) and the percentage of vacuoles containing 2, 4, 8, 16, and 32 parasites at 30 hpi (**b**) and 42 hpi (**d**) are shown. The data represent the mean ± SD for three independent experiments. * P < 0.05

The average number of *N. caninum* tachyzoites per vacuole in caprine EECs was significantly increased by transfection with chi-miR-146a mimics at both 30 hpi (Fig. 6a, b) and 42 hpi (Fig. 6c, d), while the opposite effect was found at these two time points when a chi-miR-146a inhibitor was used during transfection (Fig. 6). These findings indicated that the propagation of *N. caninum* tachyzoites in caprine EECs was promoted by chi-miR-146a.

## Discussion

Dysregulation of miRNA expression has been reported for several tissues/organs (e.g. ovary [45], liver [46], pituitary [47], lung [48]) and types of cells (e.g. skeletal muscle satellite cells [49], skin fibroblast cells [50], Leydig cells [51], blood leukocytes and milk somatic cells [52], intramuscular preadipocytes [53], endometrial epithelium cells [54]) in physiological and pathological processes in goats, including in response to infectious diseases. For example, the expression of 316 DEmiRNAs was found in peripheral blood mononuclear cells of goats infected with peste des petits ruminants virus, and most of the predicted targets of these DEmiRNAs were found to be involved in immune escape [55]. *Brucella melitensis* M5-90 infection induced 777 DEmiRNAs in goat fibroblasts, and targets for these DEmiRNAs were predicted to function in immune responses (e.g. cytokine-cytokine receptor interaction, natural killer cell-mediated cytotoxicity and Toll-like receptor signaling pathway, TNF signaling pathway, MAPK signaling pathway and JAK/STAT signaling pathway) [56]. Additionally, *Fasciola gigantica*-derived excretory-secretory products were found to induce 30 DEmiRNAs of goat peripheral blood mononuclear cells, and predicted targets for these DEmiRNAs were significantly enriched in biological processes (e.g. cell differentiation, cell development and regulation of nervous system development) and the TGF-β signaling pathway [57]. These findings suggested that miRNAs have significant biological roles in pathophysiological processes.

The uterus is the reproductive organ in which the fetus develops in placental mammals [58]. Using RNA-seq, 578 and 464 miRNAs were identified in the endometrium of Xinong Saanen dairy goats and Chuanzhong black goats, respectively [59, 60]. A total of 143 miRNAs were differentially expressed during embryo implantation in Xinong Saanen dairy goats [59], and 33 DEmiRNAs were identified in goats on day 16 of pregnancy that were not present in non-pregnant goats on day 16 of the estrous cycle [60]. Additionally, 434 miRNAs were identified in the extracellular vesicles of Chuanzhong black goat uterine fluid, with 106 DEmiRNAs identified during peri-implantation [61]. To elucidate the pathophysiological events that take place in the uterus during infection with *N. caninum*, in the present study we determined the expression profiles of miRNAs in caprine EECs infected with *N. caninum* at 24 hpi and 48 hpi. A total of 1239 miRNAs were identified in the caprine EECs, of which 891 are novel miRNAs, and thus our findings greatly enhance the miRNA database with respect to information on goats.

In the present study, *N. caninum* infection induced 18 and 79 DEmiRNAs at 24 hpi and 48 hpi, respectively. Functional analysis showed that most of the targets of these DEmiRNAs were significantly enriched in immune-related signaling pathways (e.g. Fc gamma R-mediated phagocytosis, Toll-like receptor signaling pathway, TNF signaling pathway, TGF-β signaling pathway, MAPK signaling pathway). Of these DEmiRNAs, downregulated miR-193b-5p has been found to target HMGA2 to inhibit 5-fluorouracil-induced apoptosis through the MAPK signaling pathway [62]. Downregulated miRNA-455-3p promoted TGF-β signaling and inhibited the development of osteoarthritis by targeting PAK2 [63]. Furthermore, downregulated miR-361-3p was predicted to regulate the expression of MAP3K8 through the TNF signaling pathway in our study, and MAP3K8 has been reported to promote angiogenesis and inhibit inflammation with the participation of the tumor necrosis factor-α (TNF-α) signaling pathway [64]. Interestingly, C57BL/6 mice infected with *N. caninum* exhibited higher mortality associated with inflammatory lesions and increased parasite burden in the brain through TNF-TNFR1 signaling [65]. In addition, in our study, downregulated miR-128-5p was predicted to activate MKNK2 through the MAPK signaling pathway. Previous studies showed that *N. caninum* evaded antigen presentation from bone marrow-derived macrophages by activating p38 MAPK [66], and extracellular vesicles secreted by *N. caninum* regulated the cytokine expression of bone marrow-derived macrophages through Toll-like receptor 2 and MAPK signaling pathways [67]. Furthermore, a 14-3-3 protein of *N. caninum* could induce effective immune responses and stimulate cytokine expression by activation of the MAPK signaling pathway [68]. These findings suggest that these DEmiRNAs play important roles in immune defense or immunopathogenesis during *N. caninum* infection.

Additionally, the functional analysis also showed that targets of the DEmiRNAs were significantly enriched in metabolism-related pathways (e.g. lysine degradation, insulin signaling pathway, AMPK signaling pathway, Rap1 signaling pathway, calcium signaling pathway). Among these DEmiRNAs, downregulated miR-128-5p was predicted in our study to regulate the expression of inhibitor of DNA-binding 1 through the Rap1 signaling pathway; inhibitor of DNA-binding 1 has been reported to play a key role in cell adhesion in neural stem cells through the preservation of Rap1 signaling [69]. Downregulated miR-877-3p was predicted in our study to regulate the expression of sirtuin 1 (SIRT1) through the AMPK signaling pathway. A previous study showed that SIRT1 could suppress lipogenesis through the AMPK signaling pathway [70], and our group found that SIRT1 promoted cell autophagy and intracellular proliferation of *N. caninum* tachyzoites in caprine EECs through inducing mitochondrial dysfunction [71]. These findings indicate that these DEmiRNAs play a significant role in the interaction between the host and *N. caninum* through metabolic regulation.

Interestingly, five of the DEmiRNAs (miR-146a, miR-155-5p, miR-155-3p, miR-17-5p, miR-20a-5p) in the present study were also found to be dysregulated during *T. gondii* infection in previous studies [29, 72–74]. Of these, miR-146a, miR-155-5p and miR-155-3p were upregulated during infections with two different protozoan parasites [72–74], but the opposite was found for miR-17-5p [29] and miR-20a-5p expression [29]. Notably, miR-146a has been identified as a microRNA fingerprint associated with *Toxoplasma* persistence in the host brain [72]. Previous studies showed that miR-146a is a negative regulator of innate immune response [75], and is associated with inflammatory immune responses (e.g. systemic lupus erythematosus [76], osteoarthritis [77], rheumatoid arthritis [78]), tumors (e.g. colorectal cancer [79], breast cancer [80], gastric cancer [81], lung cancer [82]), angiogenesis [83, 84], apoptosis [85], and autophagy [86]. In addition, miR-146a was reported to affect gene expression through various signaling pathways, e.g. TNF-α, NF-κB, MEK-1/2 and JNK-1/2 [82]. In our study, chi-miR-146a was upregulated and promoted the propagation of *N. caninum* tachyzoites in caprine EECs, suggesting that this miRNA plays a role in the intracellular survival of *N. caninum* tachyzoites in host cells. Thus, the regulatory mechanism of chi-miR-146a should be further studied in future work.

## Conclusions

*N. caninum* induced significant differential expression of miRNA in caprine EECs at 24 hpi and 48 hpi, and functional analysis showed that the DEmiRNAs may play significant roles in the interaction between *N. caninum* and caprine EECs. Upregulated chi-miR-146a promoted the propagation of *N. caninum* tachyzoites in caprine EECs. These findings provide fundamental data for further elucidation of the significance of ncRNAs in host defense and pathogenesis during *N. caninum* infection.

## Supplementary Information

**Additional file 1: Data S1.** The sequences and annealing temperatures of the primers for the quantitative real-time polymerase chain reaction of selected differentially expressed (DE) microRNAs (miRNAs).

**Additional file 2: Data S2.** The statistics of the reference genomes matched to *Capra hircus.*

**Additional file 3: Data S3.** The sequences and lengths of the identified miRNAs.

**Additional file 4: Data S4.** All the DEmiRNAs.

**Additional file 5: Data S5.** The predicted target mRNAs for the DEmiRNAs.

**Additional file 6: Data S6.** Gene Ontology (GO) enrichment analysis of targets for all the DEmiRNAs.

**Additional file 7: Data S7.** Kyoto Encyclopedia of Genes and Genomes (KEGG) enrichment analysis of targets for all the DEmiRNAs.

## Data Availability

The datasets supporting the findings of this article are included within the article and its additional files.

## Abbreviations

AMPK: AMP-activated protein kinase
BP: Biological process
CC: Cellular composition
DE: Differentially expressed
EECs: Endometrial epithelial cells
FBS: Fetal bovine serum
GO: Gene Ontology
hpi: Hours post-infection
KEGG: Kyoto Encyclopedia of Genes and Genomes
MAPK: Mitogen-activated protein kinase
MF: Molecular function
miRNAs: MicroRNAs
mRNA: Messenger RNA
ncRNAs: Non-coding RNAs
nt: Nucleotide
qRT-PCR: Quantitative real-time polymerase chain reaction
RNA-seq: RNA sequencing
SIRT1: Sirtuin 1
TGF: Transforming growth factor
TNF: Tumor necrosis factor
